# *Klebsiella pneumoniae* Carbapenemase–Producing Enterobacterales Infection and Colonization in a Long-Term Care Facility — Ontario, Canada, May 2024–January 2025

**DOI:** 10.15585/mmwr.mm7519a1

**Published:** 2026-05-21

**Authors:** Mehdi Aloosh, Handreen Mohammed Saeed, Laura F. Mataseje

**Affiliations:** ^1^Windsor-Essex County Health Unit, Windsor, Ontario, Canada; ^2^Department of Health Research Methods, Evidence, and Impact, McMaster University, Hamilton, Ontario, Canada; ^3^National Microbiology Laboratory, Winnipeg, Manitoba, Canada.

**Summary**

**What is already known about this topic?**

Carbapenemase-producing Enterobacterales (CPE), particularly *Escherichia coli* CPE, can spread in long-term care facilities (LTCFs) through asymptomatic persons who are carriers, staff members, shared spaces, and device use, causing difficult-to-treat infections among residents with underlying medical conditions and those with indwelling devices.

**What is added by this report?**

An 8-month outbreak of CPE in an Ontario, Canada, LTCF affected five residents; whole genome sequencing identified clonal spread of *E. coli* sequence type 131 and a closely related, approximately 62-kb IncN1 *bla*_KPC-3_ plasmid across *E. coli* and *Klebsiella* *pneumoniae*, indicating dual organism- and plasmid-mediated transmission.

**What are the implications for public health practice?**

Adoption of LTCF risk-based admission screening, clear interfacility transfer communication, prompt implementation of contact precautions for persons who are documented as asymptomatic carriers, routine hand hygiene, compliance with infection prevention and control practices, and focused environmental controls coupled with antimicrobial stewardship could help prevent future outbreaks.

## Abstract

The emergence of antibiotic-resistant organisms is a substantial public health concern because these organisms can cause severe, difficult-to-treat infections and spread easily in congregate settings. In January 2025, an outbreak of carbapenemase-producing Enterobacterales (CPE) infection and colonization was identified in a long-term care facility (LTCF) in Ontario, Canada, after a resident’s urine culture grew *Escherichia coli* carrying the *Klebsiella pneumoniae* carbapenemase (KPC) gene. An investigation by the local public health agency in Windsor, Ontario, revealed that the source of infection was likely another patient with KPC-producing *K. pneumoniae* infection who had been admitted to the LTCF 1 year earlier without appropriate infection prevention and control precautions. The epidemiologic link between the two patients triggered unitwide screening. Three additional asymptomatic carriers who had not previously received CPE testing were identified during point-prevalence screening. All five affected residents were older adults with multiple comorbidities (including patients with frequent antibiotic use or an indwelling device such as a urinary catheter) who lived in the same unit and shared multiple potential exposure sources that likely contributed to the spread. Potential sources included overlapping staff member assignments, use of a common-area bathroom, and shared dining facilities. Whole genome sequencing confirmed the outbreak. Public health responses included rapid implementation of contact precautions, staff member cohorting, enhanced environmental cleaning, ensuring compliance with enhanced infection prevention and control practices, and risk communication with facility staff members, which halted further spread; evidence does not support the use of antimicrobials or other methods to decolonize asymptomatic CPE carriers. The outbreak highlighted the need for risk-based admission screening of LTCF residents, consistent infection-prevention practices, and antibiotic stewardship. Strengthening these practices can help prevent prolonged undetected transmission and reduce the incidence of CPE infection in congregate care settings.

## Introduction

In January 2025, the public health agency in Windsor, Ontario, received notification of a confirmed case of carbapenemase-producing Enterobacterales (CPE) infection in a patient with an unknown history of CPE colonization. The organism was detected in a urine culture from a long-term care facility (LTCF) resident (i.e., patient A, the index patient) who had a history of recurrent urinary tract infections and repeated antibiotic use. The isolate was *Escherichia coli* carrying the *Klebsiella pneumoniae* carbapenemase (KPC) gene. Further investigation determined that patient A had shared a room since May 2024 with patient B (i.e., the primary patient), whose urine culture at the time of hospital-to-LTCF transfer grew KPC-producing *K. pneumoniae.* However, contact precautions were not implemented for patient B on admission to the LTCF; this patient had multiple comorbidities, was restricted to the bed, used indwelling devices (including a gastrostomy tube and urinary catheter), and had received frequent treatment for urinary tract infections. An outbreak investigation was undertaken to identify the source of the infection and the extent of the outbreak.

## Investigations and Results

The LTCF included approximately 120 beds on two floors (approximately 60 beds per floor). Each floor was divided into two units, with approximately 30 beds per unit. Identification of the epidemiologic link between the two residents with CPE infection prompted an outbreak investigation, including point-prevalence screening. This outbreak investigation was conducted under the authority of Ontario’s Health Protection and Promotion Act as part of the mandate of the Windsor-Essex County Health Unit. As such, it did not require review by the research ethics board. All data were de-identified before analysis and reporting.

### Point-Prevalence Screening

**Identification of additional residents with colonization.** Four rounds of point-prevalence screening (collection of rectal swabs) were conducted in the LTCF during a 3-week period. The first round included all residents of the unit where the two residents with CPE infection lived and identified three additional residents in the same unit with colonization (i.e., patients C, D, and E), none of whom had previously received testing for CPE. None of these patients reported recent hospitalization or receipt of health care outside Canada. The point-prevalence testing also determined that patient B was colonized with KPC-producing *E. coli*. This patient initially received a diagnosis of KPC-producing *K. pneumoniae* infection in 2024.

**Characteristics of residents colonized with CPE.** All five persons colonized with CPE were aged ≥65 years and had multiple chronic conditions, including diabetes (three patients), dementia (three), cardiovascular disease (two), and renal disease (one), and all required substantial assistance for activities of daily living; three residents had recently used antimicrobials, and one had an indwelling device. The investigation revealed that four of the residents had socialized together in the LTCF for an extended period before the outbreak investigation. The organism colonizing all five persons was *E. coli* carrying the KPC gene. However, patient B’s initial (2024) urine culture yielded *K. pneumoniae* carrying the KPC gene, suggesting potential plasmid-mediated gene transfer between the two bacterial species in patient B.

**Whole genome sequencing.** A total of six isolates collected from the five affected residents, including five *E. coli* and one *K. pneumoniae,* were sent to the National Microbiology Laboratory in Winnipeg, Manitoba, for whole genome sequencing using the Illumina NextSeq 2000 platform. Four *E. coli* isolates belonged to sequence type (ST) 131 and differed by a range of one through nine single nucleotide variants (SNVs)*; this finding supported clonal transmission within the LTCF unit. All six isolates carried the *bla*_KPC-3_ gene on a closely related (approximately 62-kb) IncN1 plasmid. Plasmid phylogeny showed tight clustering, consistent with plasmid‑mediated transmission among residents and across species, even though only the four *E. coli* ST131 isolates clustered tightly at the chromosomal level. (Table).

**TABLE:** Characteristics of six carbapenemase-producing Enterobacterales isolates identified during an outbreak at a long-term care facility — Ontario, Canada, May 2024–January 2025

Patient	Test date	Bacteria isolated	Sequence type*	KPC allele^†^	Plasmid type^§^
B (Primary)	2024	*Klebsiella pneumoniae*	556	KPC-3	IncN1
B (Primary)	2025	*Escherichia coli*	131	KPC-3	IncN1
A (Index)	2025	*Escherichia coli*	131	KPC-3	IncN1
C	2025	*Escherichia coli*	69	KPC-3	IncN1
D	2025	*Escherichia coli*	131	KPC-3	IncN1
E	2025	*Escherichia coli*	131	KPC-3	IncN1

**Abbreviation:** KPC = *Klebsiella pneumoniae* carbapenemase.

* Multilocus sequence type assigned to each isolate based on allelic profiles of housekeeping genes using multilocus sequence typing analysis. Sequence type reflects the clonal lineage of the organism.

^†^ Specific variant of the *bla*_KPC_ gene identified in each isolate (e.g., KPC-2 and KPC-3), indicating the carbapenemase allele responsible for carbapenem resistance.

^§^ Plasmid incompatibility group or plasmid type carrying the *bla*_KPC_ gene, as determined by plasmid typing analysis (e.g., IncF, IncN, and IncX3). The plasmid is the genetic vehicle responsible for horizontal transmission of the resistance gene.

## Public Health Response

**Infection prevention and control inspection.** After identification of patient B as an asymptomatic carrier, the Windsor-Essex County Health Unit conducted an on-site inspection of the LTCF in January 2025. The infection prevention and control (IPC) assessment identified several areas for concern, including 1) failure to maintain contact precautions for patient B despite documented KPC infection at the time of the patient’s May 2024 transfer to the LTCF, 2) the absence of a structured admission screening protocol for residents at increased risk for carrying antimicrobial-resistant organisms, and 3) lack of adherence to hand hygiene and personal protective equipment practices among staff members. Use of a common-area bathroom by residents in the affected unit was also noted and considered a plausible contributor to transmission.

**Outbreak control measures.** The LTCF implemented unitwide outbreak control measures, including contact precautions and private rooms with dedicated bathrooms for colonized residents, cohorting of staff members, restriction of visitors to essential visits, suspension of group dining, and suspension of group activities in the affected unit. Enhanced environmental cleaning with dedicated equipment was instituted, and a scheduled sink-drain cleaning protocol and plumbing modification were implemented to mitigate splash back after residents washed their hands, given concern about the plumbing-associated spread of organisms from patient B to patient A. Windsor-Essex County Health Unit staff members provided on-site training to LTCF staff members about CPE transmission, hand hygiene, personal protective equipment use, and other IPC practices and increased IPC audit frequency to ensure compliance.

Patients involved in this outbreak did not receive retesting. Evidence does not support the use of antimicrobials or other methods to decolonize asymptomatic CPE carriers (*1*) because 1) spontaneous clearance occurs in approximately 50%–55% of patients after 6–12 months (*2*); 2) administration of antibiotics to persons who are asymptomatic carriers could lead to proliferation of organisms with increased antibiotic resistance (*3*); 3) antibiotic treatment can disrupt the microbiome, which could paradoxically increase susceptibility to further colonization and infection; and 4) studies demonstrate a limited clinical benefit of decolonization (*3*). Therefore, management of this outbreak was limited to IPC measures, per Canadian provincial guidance (*4*).

**Additional point-prevalence screenings.** A second round of point-prevalence screening was performed 1 week after the first round of testing in the affected unit and the adjacent unit on the same floor. One additional resident received a positive test result, for a New Delhi metallo-β-lactamase–producing CPE infection. The patient was admitted to the LTCF with this diagnosis in 2024. This resident’s care was managed with contact precautions in a private room, and the case was not considered part of the outbreak because the organism and timeline were unrelated. Two additional point-prevalence screening rounds were conducted at weekly intervals after the second round; no further transmission was identified. The weekly screening was discontinued, and the outbreak was declared over; IPC measures were continued.

## Discussion

This investigation documented intrafacility transmission of KPC-producing Enterobacterales among residents of a single LTCF unit in Canada during approximately 8 months (May 2024–January 2025). Multiple converging factors likely facilitated the spread, including the presence of a person with documented colonization (i.e., patient B) admitted to the LTCF without implementation of contact precautions, overlapping staff member assignments, shared dining facilities, and use of a common-area bathroom. The residents were older adults with multiple comorbidities, indwelling devices, and frequent antimicrobial use. Previous research has identified a high prevalence of CPE among LTCF residents (*5*) linked to similar risk factors (*6*).

Genomic evidence strengthened the epidemiologic findings in this outbreak. Clustering of *E. coli* ST131 isolates differing by only one to nine SNVs is consistent with recent clonal transmission. Detection of *bla*_KPC-3_ on closely related IncN1 plasmids across *E. coli* and *K. pneumoniae* isolates supports plasmid-mediated spread, which can propagate resistance across species even when organism-to-organism transmission is limited. Together, these findings indicate dual transmission pathways (i.e., via the organism and the plasmid) that can sustain and amplify CPE within congregate care settings.

The public health response, including the rapid institution of contact precautions, staff member cohorting, activity restrictions, education, compliance with enhanced IPC practices, and environmental interventions, including sink-drain procedures, was temporally associated with the interruption of transmission, as evidenced by three subsequent negative screening rounds after identification of the residents with colonization. Although environmental sampling was not conducted, mitigation measures, such as plumbing modifications to reduce the risk for splash back after residents washed their hands, were reasonable.

### Limitations

The findings in this report are subject to at least two limitations. First, point-prevalence screening was restricted to the affected unit and one adjacent unit, which might have resulted in an underestimate of the number of persons colonized within the LTCF. Conducting multiple rounds of facilitywide point-prevalence testing is resource-intensive; therefore, a stepwise approach was adopted, expanding testing to other units only when further positive results were reported in the tested unit. Once no additional cases were identified, testing was not expanded further. Second, environmental testing for CPE is not routinely performed by the Canadian provincial public health laboratory and was not conducted. In addition, the transmission pathway involved mobile patients, and enhanced cleaning protocols were already in place. These factors precluded direct assessment of surface or plumbing contamination.

### Implications for Public Health Practice

This outbreak highlights four strategies for preventing introduction and spread of antibiotic-resistant organisms in LTCFs: 1) adoption of risk-based screening (e.g., recent hospitalization or interfacility transfer, previously identified patients who are carriers of antimicrobial-resistant organisms, and patients using indwelling devices) so that patients who are carriers can be identified when they are admitted to LTCFs and timely precautions can be implemented (*7*,*8*); 2) implementing contact precautions for known carriers when they are admitted or transferred from another facility, with clear signage, dedicated equipment when feasible, and prompt staff member education to help support adherence; 3) detailed cleaning protocols for shared bathroom facilities, attention to sink-to-patient splash zones, and, where feasible, provision of private bathrooms for carriers to help reduce risk for transmission; and 4) use of antibiotic stewardship programs, because frequent and prolonged antibiotic use is a recognized driver of acquisition and persistence of resistant organisms. Ensuring antibiotic stewardship programs that review indications and durations and consider the most narrow-spectrum antibiotics, particularly for recurrent urinary tract infections, can reduce transmission in LTCFs.

## Footnotes

* SNVs are defined by single DNA variations in a genome; an SNV refers to any single nucleotide change, even if it occurs only once.
